# The response of zooplankton network indicators to winter water warming using shallow artificial reservoirs as model case study

**DOI:** 10.1038/s41598-023-45430-7

**Published:** 2023-10-21

**Authors:** Anna Maria Goździejewska, Marek Kruk

**Affiliations:** 1 Faculty of Geoengineering, University of Warmia and Mazury in Olsztyn, Oczapowskiego 5, 10-719 Olsztyn, Poland; 2 Faculty of Mathematics and Computer Science, University of Warmia and Mazury in Olsztyn, Słoneczna 54, 10-710 Olsztyn, Poland

**Keywords:** Environmental sciences, Limnology

## Abstract

To predict the most likely scenarios, the consequences of the rise in water surface temperature have been studied using various methods. We tested the hypothesis that winter water warming significantly alters the importance and nature of the relationships in zooplankton communities in shallow reservoirs. These relationships were investigated using network graph analysis for three thermal variants: warm winters (WW), moderate winters (MW) and cold winters (CW). The CW network was the most cohesive and was controlled by eutrophic Rotifera and Copepoda, with a corresponding number of positive and negative interspecific relationships. An increase in water temperature in winter led to a decrease in the centrality of MW and WW networks, and an increase in the importance of species that communicated with the highest number of species in the subnetworks. The WW network was the least cohesive, controlled by psammophilous and phytophilous rotifers, and littoral cladocerans. Adult copepods were not identified in the network and the importance of antagonistic relationships decreased, indicating that the WW network structure was weak and unstable. This study can serve as a model for generalisations of zooplankton community response to the disappearance of long winter periods of low temperatures, as predicted in global climate change projections.

## Introduction

The study of water temperature variability and its effects on ecosystem structure, stability and functions in the era of global warming has become increasingly important in recent years. Temperature is one of the key factors regulating life processes in the aquatic environment, and it influences gas exchange, the degree of saturation and the rate of nutrient and organic matter cycling in water^1,2^. Water temperature gradients lead to changes in biocoenosis composition and food chain structure^3–6^. In temperate climates, water bodies are influenced by seasonal variations in air temperature and solar radiation, which lead to different temperature regimes^1^. Shallow waters are particularly vulnerable to atmospheric fluctuations, as air temperature and the mechanical effects of wind lead to frequent changes in thermal and oxygen profiles^7^.

Fluctuations and/or permanent changes in the thermal profile of surface waters have been widely associated with the discharge of industrial cooling water^8–12^. The effect of winter warming in the reservoirs studied is caused by the indirect artificial supply of geothermal water from deeper deposits exposed by lignite mining. The impact of local geothermal springs has been studied less frequently and mostly focuses on their local use for therapeutic purposes (balneotherapy) or as a tourist attraction^13–16^. In general, there are few studies on the hydrobiological impacts of geothermal water entering water bodies, and the few available studies have mainly focused on tropical regions^17–19^. In recent decades, climatic factors associated with a global temperature increase, particularly in northern latitudes, have been identified as additional drivers of changes in the thermal profile of surface waters^20–22^. An increasing temperature gradient can significantly affect the structure and functioning of aquatic biocenoses in temperate climates, and the sensitivity of aquatic organisms results from evolutionary adaptations to specific thermal regimes^23–26^.

Zooplankton support important processes in aquatic ecosystems. They play key roles in the food web by linking primary producers with consumers at higher trophic levels (fish)^27–29^. Planktonic animals are the most important link in the microbial carbon cycle^30^ and they are sensitive bioindicators of changes in abiotic factors^31–33^. Due to their taxonomic and functional diversity, their different ecological strategies, their phylogenetic features, and their passive and widespread distribution in the environment, zooplankton are very useful for the development of ecosystem models and predictions, including in the context of global warming^24,34–41^. Zooplankton respond directly to water temperature at physiological (by regulating metabolic, growth and developmental processes)^42,43^ and behavioural levels (changes in distribution, population size, species composition and phenology)^9,44,45^. Water temperature, in turn, indirectly affects zooplankton communities by determining the availability and quality of food resources (mainly phytoplankton) and the intensity of fish predation^3,26,33^.

Previous research has shown that an increase in mean seasonal/annual water temperature causes similar responses in freshwater zooplankton as accelerated eutrophication. The observed responses were: an increase in total zooplankton density and biomass, changes in species composition^11,36,46,47^, the elimination of seasonal succession, including a decrease in the proportion of cold-water species in spring rotifer communities^45^, a decrease in the size of copepods and accelerated growth of cladocerans, which are characterised by small body size/low biomass^5,26,48,49^. Seasonal shifts, caused in part by early spring water warming, accelerate the development of thermophilic species and often disrupt their natural life cycle^50–52^. Particular attention has been paid to phenological changes, as the timing at which temperatures begin to rise determines reproductive success, emergence from dormancy, generation time and food availability^43,53–55^. Therefore, species-specific responses to changes in the thermal regime are directly reflected in the taxonomic structure, biomass and functional properties of zooplankton communities^25,26,54,56^. These factors affect the organisation of interspecific relationships in zooplankton networks, which consist mainly of competition and predation, and they influence successive trophic levels^44,57,58^. An analysis of the interactions between changing temperatures and zooplankton community characteristics provides valuable information for assessing the state of aquatic ecosystems and predicting future changes.

This study proposes a new, structural approach to describe the responses of zooplankton to water temperature. Due to the extensive direct and indirect role of thermals in shaping the zooplankton structure discussed above, we assumed that other physico-chemical factors of feed (inflow) waters are not the key. This assumption is confirmed by the results of our previous work about “winter warming”, using the SHAP model^39^.

Interspecific interactions were investigated using network graph analysis. A network graph model supports the identification and assessment of relationships between species based on mutualism or neutral coexistence of species in ecological guilds (positive mathematical interactions) or constraints (negative correlations) due to predation or competition^37,40,59,60^. In the network approach chosen for the zooplankton biocoenosis, the object of our study was the species structure expressed by the taxon biomass network, which refers to the whole season. We did not consider the dynamics of changes in biomass within the analysed periods and related the effects of these changes to the whole season. Studying the dynamics of changes in zooplankton biomass in weekly or monthly cycles is a separate research topic. Following Krebs^61^, we assumed that positive interactions between two taxa are correlated with an increase in their biomass as an effect of consumption guilds where independent species share resources. Negative interactions between species (their biomass), in turn, are indicative of indirect negative effects due to competition for a common food source, predation or interference competition. Moreover, following the theory of biocenoses organization by Armstrong and McGehee^62^ and Levin^63^, we assumed that the proportion of positive and antagonistic (competition and predation) interspecific relationships would be a measure of equilibrium and persistence of zooplankton communities.

The reservoirs studied were created more than 20 years ago and are fed with water with relatively stable physical and chemical parameters (including temperature). This means that the feedwater influences the planktonic biocenosis by acting as a permanent environmental filter, rather than a temporary disturbance. We have assumed and showed that the variability of water temperature in the reservoirs studied is the most important physico-chemical factor distinguishing these waters. Therefore, the situation described can serve as a model for generalisations of the response of the zooplankton community of inland waters to the disappearance of long winter periods of low temperatures due to climate change, as predicted in climate change projections for Europe^20^ and at the global level^22^. Global warming will not only affect average temperatures, but also increase the frequency, intensity and duration of warm periods. Therefore, the impact of temperature variability on the structure, stability and functions of ecological communities is an important consideration^20,64^. A better understanding of the responses of zooplankton communities to long-term environmental changes is crucial for predicting the responses of freshwater ecosystems to global climate change.

We hypothesised that the water temperature gradient significantly affects the growth and competitive balance of zooplankton species, i.e. the importance of individual taxa and their interactions that determine the cohesion of the network. We hypothesised that the importance and strength of interactions between zooplankton species, encompassing an equal number of positive and antagonistic biocenotic relationships, should be highest in unchanged thermal regimes (cold winters), where zooplankton taxa should form a cohesive central network. In turn, higher winter water temperatures and flattening of the annual temperature gradient should correlate with decentralisation and fragmentation of the network, weakening interactions between zooplankton species and the role of larger crustacean species and copepods.

## Results

### Environmental variables and zooplankton distribution along the thermal gradient

**Figure 1:**
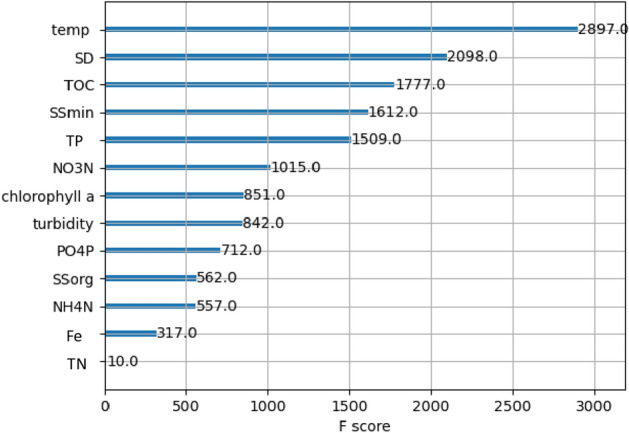
The importance of physical and chemical variables as a factor differentiating zooplankton communities in three studied reservoirs.

Significant differences in the physical parameters of the water were found between the three thermal classes. The reservoirs studied differed significantly (*P* < 0.05) in mean annual temperature and mean winter temperature, but significant variations were also observed in DO, chlorophyll* a*, TOC, TN and parameters describing suspended solids (turbidity, colour, SD, SStot) (Table 1). Water temperature significantly affected oxygen concentration, which was confirmed by a significant negative correlation between temperature and DO (*r* = − 0.555, *P* < 0.05). The XGBoost modelling showed that water temperature variability is the dominant physico-chemical differentiating factor of the three reservoirs studied, in terms the influence of thermals on zooplankton community. The F-score is definitely the highest for variable water temperature, well ahead of other important factors (SD, TOC) distinguishing the studied waters. The accuracy of this prediction is 100% for the training sub-sample and 66.7% for the test sub-sample (Fig. 1).

**Table 1:** Water quality and zooplankton parameters across the studied thermal classes (mean ± SD).

	CW		MW		WW		ANOVA *P*
	$\overline{x}$	± SD	$\overline{x}$	± SD	$\overline{x}$	± SD	
Physical and chemical parameters of water							
Temperature (°C)	13.61^a^	6.58	14.69^a^	3.91	18.49^b^	2.21	0.000
Winter temperature (°C)	5.63^a^	2.19	8.60^b^	0.76	15.35^c^	1.41	0.000
DO (mg l^−1^)	9.36^a^	1.35	9.33^a^	1.33	7.59^b^	0.97	0.000
pH	7.80	0.39	7.80	0.24	7.69	0.29	> 0.05
Chl *a* (µg l^−1^)	5.10^a^	2.86	4.32^a^	3.74	1.99^b^	4.42	0.000
TOC (mg l^−1^)	3.85^a^	3.37	1.98^b^	0.71	1.63^b^	0.62	0.000
PO_4_-P (mg l^−1^)	0.020	0.01	0.024	0.014	0.025	0.012	> 0.05
TP (mg l^−1^)	0.125	0.175	0.109	0.048	0.122	0.068	> 0.05
NO_3_-N (mg l^−1^)	0.160^a^	0.053	0.143^ab^	0.063	0.118^b^	0.045	0.032
NH_4_-N (mg l^−1^)	0.080	0.053	0.083	0.059	0.098	0.098	> 0.05
TN (mg l^−1^)	0.299^a^	0.124	0.238^ab^	0.077	0.220^b^	0.126	0.034
Turbidity (NTU)	17.30^a^	7.08	12.56^b^	7.53	9.74^b^	5.14	0.006
SD (m)	0.756^a^	0.168	0.919^a^	0.166	1.46^b^	0.32	0.000
Color (Hazen)	15.00^a^	7.09	10.04^b^	3.91	7.39^b^	2.71	0.000
SSmin (mg l^−1^)	2.95^a^	2.79	0.975^b^	0.868	1.46^b^	1.42	0.002
SSorg (mg l^−1^)	3.09	3.05	3.00	2.64	2.76	2.19	> 0.05
SStot (mg l^−1^)	6.05	4.28	3.98	3.04	4.28	2.88	> 0.05
Fe (mg l^−1^)	0.165^ab^	0.098	0.255^a^	0.204	0.138^b^	0.062	0.042
Zooplankton measures							
Biomass (mg l^−1^)	28.25^a^	33.1	0.730^b^	0.999	0.094^c^	0.064	0.000
Abundance (ind. l^−1^)	3903.1^a^	3933.5	563.2^b^	1119.2	45.03^c^	25.12	0.000
Av. number of species (ind.)	16	5	15	5	17	5	> 0.05
Total number of species (ind.)	61		74		89		–
Shannon’s biodiversity index *H*′	1.66^a^	0.37	1.62^a^	0.54	2.31^b^	0.37	0.000
Pielou’s eveness index, *J*′	0.605^a^	0.128	0.634^a^	0.206	0.824^b^	0.085	0.000

*DO* dissolved oxygen, *Chl a* chlorophyll *a*, *TOC* total organic carbon, *PO*_*4*_*-P* orthophosphate, *TP* total phosphorus, *NO*_*3*_ N nitrate, *NH*_*4*_ N ammonium, *TN* total nitrogen, *SD* Secchi depth transparency, *SStot* total suspended solids, *SSmin* inorganic suspended solids, *SSorg* organic suspended solids, *Fe* iron.

Differences in the analyzed parameters were determined by ANOVA (*P* ≤ 0.05); values with different superscripts differ significantly across reservoirs in Tukey’s HSD test.

The temperature gradient had a significant influence on the species diversity of the zooplankton. Species diversity was highest in WW (*H*′ = 2.31; *J*′ = 0.824) and significantly lower in MW and CW (*H'* = 1.62 and 1.66; *J*′ = 0.634 and 0.605, respectively; Table 1). The zooplankton community comprised 89 taxa in WW, 74 taxa in MW and 61 taxa in CW. Rotifera dominated in all thermal classes and accounted for 67% (CW) to 75% (MW) of the total zooplankton species. Greater differences were found in the taxonomic structure of Crustacea, with a dominance of Cladocera in WW (15%; 6–10.5% in the other groups) and a dominance of copepods in CW (19%; 9% in MW and 6.5% in WW). All thermal classes had 33 (26%) taxa and forms in common, with juvenile nauplii and copepodites (100% CW—92% WW and 100% CW—50% MW) and *Keratella cochlearis* (Gosse, 1851) (100% CW—50% MW) predominating, respectively (Table S1). The greatest similarities between zooplankton communities, as measured by the Jaccard index, were found between MW and WW (47.8%). The zooplankton communities in CW and WW were least similar (33.9%) (Fig. S1).

The temperature gradient resulted in significant differences in the biomass distribution of 23 (18%) zooplankton taxa (Kruskal–Wallis test, *P* < 0.05), including 15 Rotifera, 2 Cladocera, 2 Copepoda and 4 Protozoa. Most of the remaining zooplankton taxa (77%) were not represented in each thermal class (Table S1). This resulted in significant differences in the mean biomass and abundance of zooplankton in the different thermal classes. These parameters were determined at 28.35 mg l^−1^ (CW), 0.73 mg l^−1^ (MW) and 0.094 mg l^−1^ (WW) and at 3903 ind. l^−1^ (CW), 563 ind. l^−1^ (MW) and 45 ind. l^−1^ (WW), respectively (Table 1).

### Zooplankton networks differences

**Figure 2:**
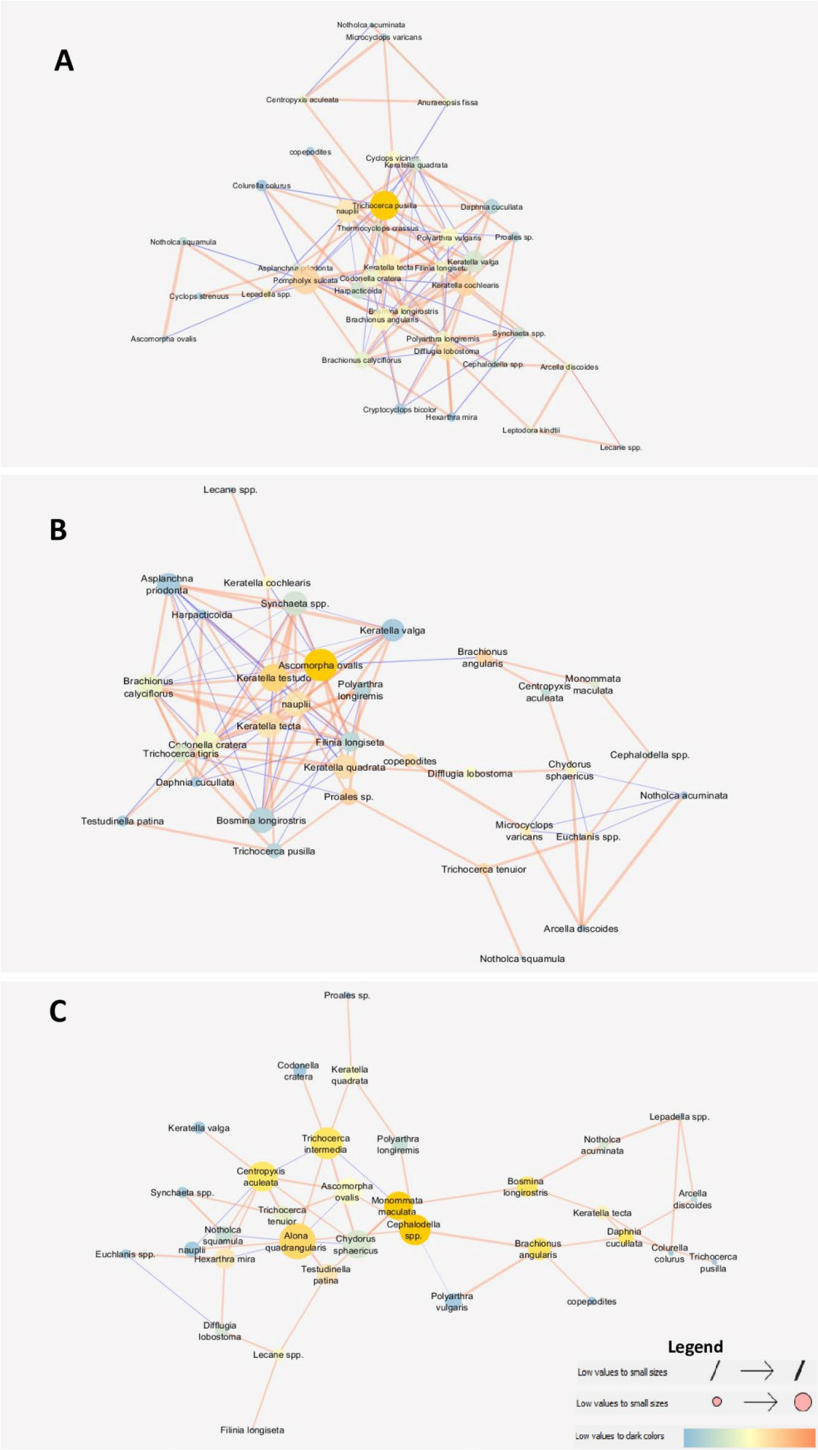
Network graph analysis of the interactions between zooplankton species in the: (**A**) CW, (**B**) MW, and (**C**) WW network, with an analysis of node closeness centrality (NCC), node betweenness centrality (NBC), and edge betweenness centrality (EBC) values. Node size is proportional to the NCC measure, node color on the blue (dark)–orange (bright) color scale is proportional to the NBC measure, and edge thickness is proportional to the EBC measure. Sign of the relationship: bright orange edges denote positive relationships between nodes, whereas dark blue edges denote negative relationships.

**Figure 3:**
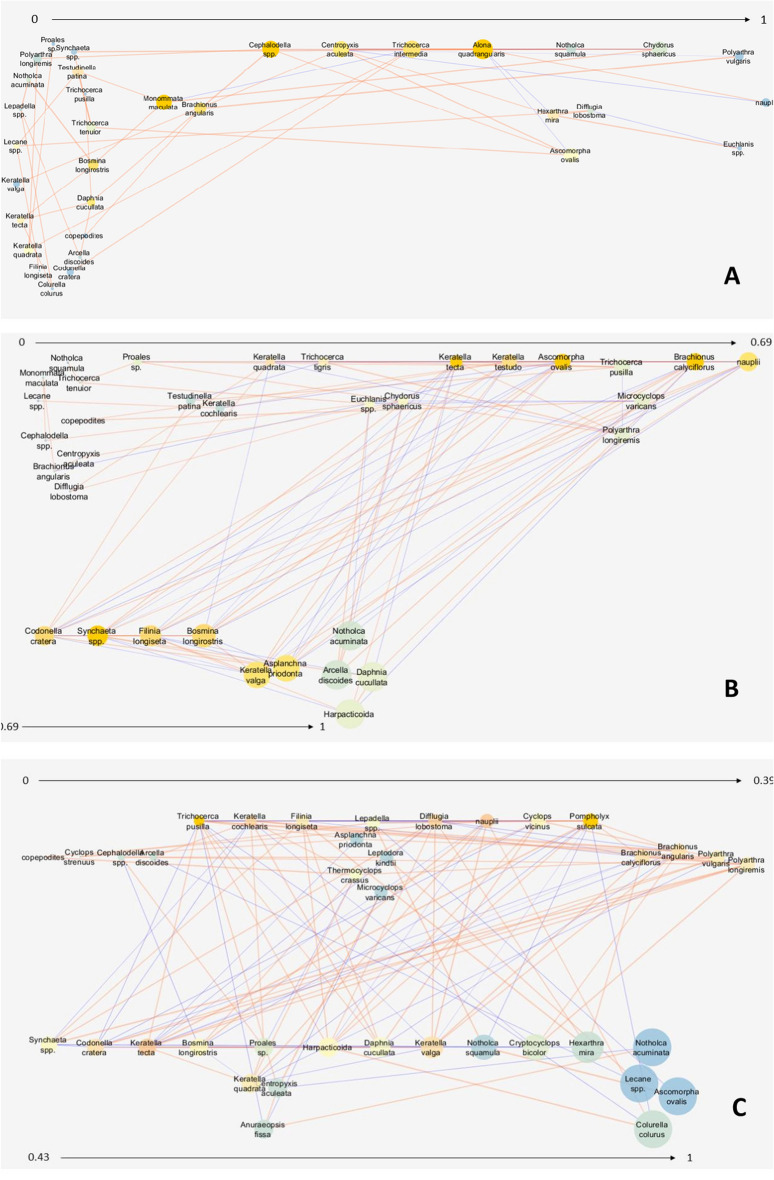
Clustering coefficient distribution in the: (**A**) WW, (**B**) MW, and (**C**) CW zooplankton network. Node size is proportional to the Clustering coefficient measure in the range 0–1, showed by arrows. For node and edges color explanations see the legend in Fig. 2.

The thermal classes compared differed in the key metrics describing the structure of the zooplankton-species interaction network. The CW network was characterised by the highest cohesion expressed by clustering (0.462), centrality metrics (0.248), the shortest paths (1406) and the highest average number of neighbours (6.32) per species (node), i.e. the number of interspecific interactions (Table 2; Fig. 2A). Density (0.191) and heterogeneity (0.617) were highest in MW, suggesting that this network was the most diverse (Table 2; Fig. 2B). The WW network was characterised by the lowest centrality (0.165) and density (0.095), and the lowest parameters of communication pathways between taxa, i.e. the shortest total sum of pathways (1056) and the longest characteristic path length (3.42), indicating the presence of taxa communicating with the lowest number of species (Table 2; Figs. 2C and 3A).

**Table 2:** General attributes of the zooplankton network in the compared thermal classes.

Attribute	Thermal class		
	CW	MW	WW
Clustering coefficient	0.462	0.437	0.191
Network centralization	0.248	0.216	0.165
Shortest paths (100%)	1406	1122	1056
Characteristic path length	2.37	2.64	3.42
Average number of neighbors	6.32	6.29	3.03
Network density	0.171	0.191	0.095
Network heterogeneity	0.518	0.617	0.577

### Interspecific interactions in zooplankton networks

Node degree centrality (NDC), defined as the number of direct links to a taxon (node), is an important indicator of interspecific relationships. The highest NDC values (more than 10 direct connections per taxon) were found in CW—*Trichocerca pusilla* (Lauterborn, 1898), *Pompholyx sulcata* Hudson, 1885, *Keratella tecta* (Gosse, 1851), and copepod nauplii, and MW—*Brachionus calyciflorus* Pallas, 1766, *K. tecta*, *Ascomorpha ovalis* (Bergendal, 1892), *Synchaeta* spp., *Ketarella testudo* (Ehrenberg, 1832), *Filinia longiseta* (Ehrenberg, 1834), cladoceran *Bosmina longirostris* (Schoedler, 1866), and protozoan *Codonella cratera* Leidy, 1887. In WW, the cladoceran *Alona quadrangularis* (Müller, 1776; 8), the protozoan *Centropyxis aculeata* (Ehrenberg, 1832; 8) and the rotifer *Trichocerca intermedia* (Stenroos, 1898; 6) (Table 3) formed the highest number of interspecific relationships.

**Table 3:** Zooplankton taxa with the highest net attribute.

	CW				MW				WW			
	NCC	NBC	NDC	CCF	NCC	NBC	NDC	CCF	NCC	NBC	NDC	CCF
Rotifera												
*Brachionus angularis* Gosse, 1851	0.521		9			0.116			0.333	0.204	4	
*Polyatrhra vulgaris* Carlin, 1943	0.500											1
*Keratella valga* (Ehrenberg, 1834)	0.521		9		0.446		10	0.911				
*Trichocerca pusilla* (Lauterborn, 1898)	0.578	0.253	15									
*Keratella cochlearis* (Gosse, 1851)	0.521	0.123	10									
*Pompholyx sulcata* Hudson, 1885	0.569	0.127	13									
*Keratella tecta* (Gosse, 1851)	0.552		11		0.465		13			0.103		
*Keratella testudo* (Ehrenberg, 1832)					0.478	0.164	11					
*Brachionus calyciflorus* Pallas, 1766			9		0.465		13					
*Keratella quadrata* (Müller, 1786)					0.458	0.106	8					
*Synchaeta* spp*.*					0.458		12					
*Asplanchna priodonta* Gosse, 1850					0.446		10	0.911				
*Proales* sp*.*						0.123						
*Filinia longiseta* (Ehrenberg, 1834)							11					
*Ascomorpha ovalis* (Bergendal, 1892)				1	0.507	0.205	13		0.360		4	
*Trichocerca intermedia* (Stenroos, 1898)									0.386	0.203	6	
*Cephalodella* spp*.*									0.381	0.265	5	
*Monommata maculata* Harring and Myers, 1924									0.368	0.254	4	
*Hexarthra mira* (Hudson, 1871)				0.833					0.327	0.119	4	
*Lecane* spp.				1								
*Colurella colurus* (Ehrenberg, 1830)				1								
*Euchlanis* spp.												1
*Notholca acuminata* (Ehrenberg, 1832)				1				1				
Crustacea												
*Alona quadrangularis* (Müller, 1776)									0.410	0.244	8	
*Chydorus sphaericus* (Müller, 1776)									0.368		4	0.666
*Bosmina longirostris* (Müller, 1785)							11	0.818		0.195		
*Daphnia cucullata* (Sars, 1867)								1		0.168	4	
Harpacticoida								1				
Nauplii	0.529		11				10					1
Protozoa												
*Codonella cratera* (Leidy, 1887)			9				11					
*Centropyxis aculeata* (Ehrenberg, 1832)									0.376	0.146	8	
*Arcella discoides* (Ehrenberg, 1843)								1				
*Difflugia lobostoma* Leidy, 1879			10									

*NCC* node closeness centrality, *NBC* node betweenness centrality, *NDC* node degree centrality, *CCF* clustering coefficient.

**Figure 4:**
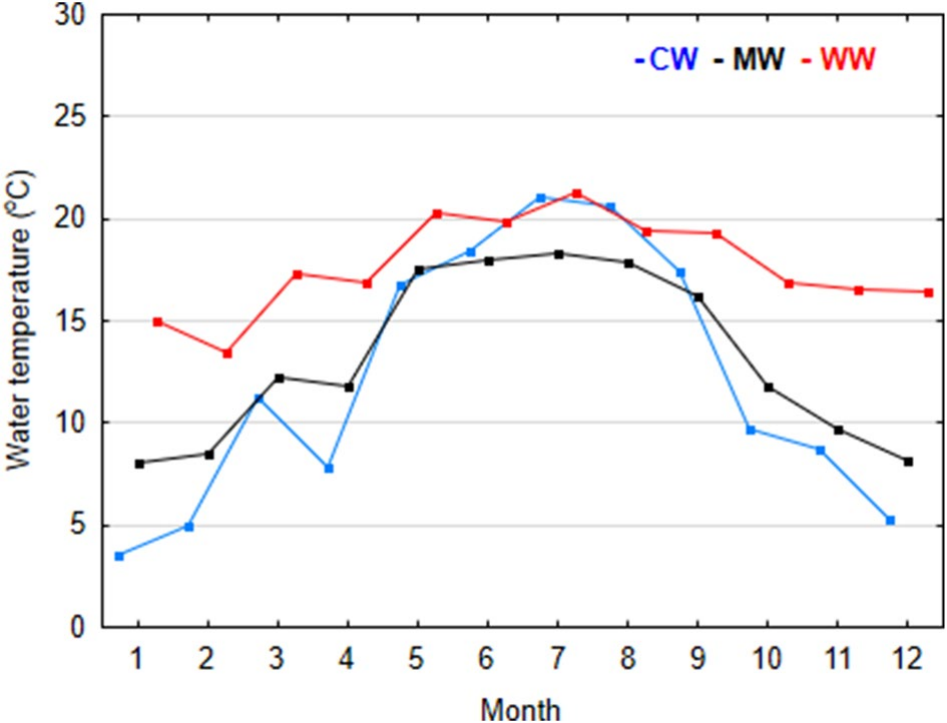
Mean monthly water temperature in the analyzed thermal classes in 2014–2016.

The strongest and most numerous interspecific relationships were formed in MW. Positive relationships with the highest values of the correlation coefficient were formed between *Polyarthra longiremis* Carlin, 1943 and *Trichocerca tigris* (Müller, 1786; 0.961), *B. longirostris* and *A. ovalis* (0.932), and *Asplanchna priodonta* Gosse, 1850 and *Keratella valga* (Ehrenberg, 1834; 0.907), while negative relationships existed between *B. calyciflorus* and *Synchaeta* spp. (− 0.821), *B. calyciflorus* and *K. valga* (− 0.754), and *B. longirostris* and *Keratella testudo* (− 0.701). Under extreme conditions, both positive and negative correlations were slightly weaker and were observed between *Notholca squamula* (Müller, 1786) and *A. ovalis* (0.837; CW), *Brachionus angularis* Gosse, 1851 and *Polyarthra vulgaris* Carlin, 1943 (0.779; WW), *B. calyciflorus* and *K. tecta* (− 0.671; CW), and *Cephalodella* spp. and *P. vulgaris* (− 0.582; WW) (Figs. 2, 3, 4; Table S2).

Node closeness centrality (NCC), which ranks nodes based on their distance from other nodes and identifies nodes whose effect spreads rapidly to most nodes in the network, decreased with an increase in temperature (CW—NCC > 0.5; MW—NCC > 0.4; WW—NCC > 0.3). The number of taxa with the above NCC values was similar in each thermal class (8, 8 and 9, respectively), but the species composition of these groups differed by 75–88% (Table 3). Rotifers *T. pusilla* and *P. sulcata* stood out with the highest centrality attribute values in CW (Fig. 2A). The highest centrality attribute values were observed for *A. ovalis* and *K. testudo* in MW, and for the cladoceran *A. quadrangularis* and the rotifer *T. intermedia* in WW (Fig. 2B,C).

Taxa in the WW network played a more important role in network cohesion, as measured by node betweenness centrality (NBC), than taxa in other thermal classes, as this attribute favours taxa that connect to sub-networks (clusters). Thus, when a network is less cohesive and more fragmented, taxa (nodes) that communicate with other network clusters play a more important role than taxa within the network. The phytophilous taxa *Cephalodella* spp. and *Monommata maculata* Harring and Myers, 1924 were characterised by the highest NBC values (> 0.250) in the WW network, but high betweenness centrality values (> 0.200) were also found for *A. quadrangularis*, *B. angularis* and *T. intermedia*. The most cohesive and central network (CW) mainly favoured *T. pusilla* (0.253), while the most heterogeneous network (MW) mainly favoured *A. ovalis* (0.205) (Table 3).

The network MW was the most heterogeneous and contained taxa with a high tendency to cluster. These taxa were connected to neighbours by the highest number of links (CCF > 0.9) and they represented all higher-ranking groups: Rotifera (*Notholca acuminata* Ehrenberg, 1832, *K. valga*, *A. priodonta*), Cladocera (*Daphnia cucullata* Sars, 1862), Copepoda (Harpacticoida), and Protozoa (*Arcella discoides* Ehrenberg, 1843). The taxa with the lowest number of connections also formed a large cluster (CCF < 0.2) (Figs. 2B and 3B).

The CW network was the most centralised and contained a much larger number of interspecific links with moderate values and no links with extreme values (CCF = 0 or 1) (Fig. 3C). The CW network was characterised by the highest CCF value and the highest cohesion (Table 2; Fig. 2 A).

## Discussion

Various methods and research hypotheses have been proposed to study and predict changes in plankton biocoenoses under the influence of increasing water temperatures^39,41,45,56,64–66^. Graph theory opens up new possibilities for analysing the structure of zooplankton networks across temperature gradients by focusing on biomass distribution as an indicator of interspecific interactions. In ecosystem ecology, all interspecific relationships involve the flow of energy (biomass), and the nature and strength of these processes are largely dependent on changes in temperature and solar radiation^67^. Temperature is a physical factor that alters the flow and conversion of energy in water, and the thermal gradient determines the intensity and direction of these processes^1,65^. The relationship between water temperature, energy flux from the atmosphere and the functioning of the aquatic biocoenosis was the reason for our interest in the changes that the zooplankton community of the artificial reservoirs might experience under climate warming conditions.

The studied reservoirs differed mainly in the winter water temperature (the difference between CW and WW = 9.7 °C), which significantly influences the annual temperature gradient (Fig. 4). Under colder conditions (CW), more energy is needed to heat the water to a similar temperature within the same winter-summer period than under warmer conditions (WW), leading to differences in the rate of physical and chemical (element cycle) processes and in biomass accumulation^68^. As a result, due to the significant increase in water temperature after winter (by 19.2 °C in CW), the organic matter cycle in the studied CW was rapidly intensified and thermal energy was distributed throughout the water column by convection or mechanical movement (the studied reservoirs are shallow). This contributed to a rapid increase in primary production (Chl *a*). In contrast, phytoplankton developed more slowly when the water temperature gradient flattened (water temperature increased by 13.1 °C in MW and 9.2 °C in WW), algal blooms were less frequent and primary production was lower^39,68^. It is worth noting that the variability of water temperature in the studied reservoirs was the most important factor that distinguished these water bodies (Fig. 1).

When the accumulated material in heated reservoirs (especially WW) circulates continuously but less rapidly, the surface water can be colonised by macrophytes that effectively use the available nutrients (TN and TOC content decreased in MW and WW) and compete with phytoplankton for food^69^. These energy transformations create trophic conditions that exert the greatest influence on species composition and function, as well as biomass and the nature and strength of interspecific interactions in zooplankton communities^38–40^.

The natural thermal regime of surface waters (CW), typical of temperate climates, promoted the development of the most connected zooplankton network with an equal number of strong positive and negative interspecific relationships. These relationships (node centrality; NCC > 0.5) were mainly formed by rotifers with a diverse food base, i.e. detritophagous and bacteriophagous *Pompholyx sulcata* and *Brachionus angularis*, phytophagous *Keratella tecta* and *K. valga*^70^, and raptorial *Polyarthra vulgaris* and *Trichocerca pusilla*. In the CW network, these species formed numerous (NDC > 10) and strong relationships with other taxa, mostly copepods. In the CW network, the above-mentioned Rotifera nodal species were characterised by higher biomass than in warmer classes, and these taxa could be considered effective bioindicators^71,72^ of good feeding conditions (eutrophic) in the CW class. The above observation was confirmed by the highest values of primary productivity (Chl *a*), organic carbon (TOC) and nitrogen in CW compared to the other thermal classes. In the CW network, the total zooplankton biomass was more than 40 and 300 higher than in MW and WW, respectively. This result differs from predictions based on correlations between increasing water temperature and biomass accumulation in the food chain^11,47^, but it confirms that energy processes in ecosystems (their importance and impact) are related to the amplitude of water temperature.

In the coldest reservoir (CW), an increase in trophic level probably also contributed to a higher content of mineral suspension. Suspended particles form a substrate that is easily colonised by algae, bacteria and protozoa, and they contribute to the accumulation of organic matter (greater availability of food resources) and its effective cycling^40,73,74^. According to Bonecker et al.^75^, the concentration of mineral suspensions strongly correlates with the concentration of chlorophyll *a*, which is an important predictor of increased rotifer biomass and copepod diversity in lotic ecosystems. The results of the present study confirm the above observation, as copepods were represented by various species characteristic of astatic habitats and small waters (*Microcyclops varicans* Sars, 1863, *Cryptocyclops bicolor* Sars, 1863), eurytopic species (*Cyclops vicinus* Uljanin 1875, *C. strenuus* Fisher, 1851, *Thermocyclops crassus* Fisher, 1853) and benthic Harpacticoida^76^. These species were characterised by low values of centrality attributes in CW, but they contributed to the formation of valuable antagonistic (predator–prey) relationships. According to Currie^67^ and Schmitz and Trussell^77^, predatory behaviour is crucial for maintaining high interspecific cohesion, as it prevents the exclusion of competitors and the loss of species diversity.

The abundance and diversity of food resources in CW were responsible not only for the strength but also for the closeness of interspecific relationships, expressed in the highest number of shortest communication paths between taxa. A similar dependence between high primary production (organic matter), high total zooplankton biomass, short path length and high network cohesion was found by Goździejewska and Kruk^40^ in a study on environmental gradients (turbidity). Kruk et al.^38^ also found that network cohesion and the strength of interspecific interactions increased with an increase in salinity, which was correlated with trophic levels in coastal lakes.

In the present study, an increase in water temperature resulted in changes in the species composition of zooplankton communities, including those with the highest values for the centrality attribute, and similar observations were made by Richardson^24^, Alric et al.^35^ and Carter et al.^44^. Similar to CW, Rotifera also contributed most to network centrality in warmer reservoirs (MW and MM), but their ecological and functional structure was significantly altered. Few studies have investigated the responses of rotifers to changes in water temperature, pointing to their lower sensitivity, i.e. their tolerance to a wider range of temperatures^78^, and a correlation between their lower reactivity compared to crustaceans^79^. Most rotifers are eurythermal species, therefore the results of studies analysing species-specific responses, such as migration patterns in vertical temperature and feeding gradients in deep lakes^80,81^, cannot be used to formulate broad conclusions about global environmental changes. Following Obertegger and Flaim^80^, changes in water temperature affect the structure of relationships between rotifers species based on feeding and predation, which is consistent with the present results.

In the MW network, raptorial rotifers (*Ascomorpha ovalis* and *Synchaeta* spp.), rotifers that graze selected algae (*Keratella testudo*, *K. quadrata* and *Brachionus calyciflorus*) and predatory rotifers (*Asplanchna priodonta*) had the highest network centrality attributes^80,82^. At the same time, these species formed the strongest positive and antagonistic relationships, especially with the cladoceran *Bosmina longirostris*. These observations suggest lower trophic levels in MW (resulting from the energy transfer described above), including lower phytoplankton production and food availability^72^. In a study by Goździejewska et al.^72^, the chemical parameters of sediments in the PN reservoir (represented by the MW class in this study) led to a decrease in phosphorus concentration in the water. Lower phosphorus levels inhibited the development of more demanding phytoplankton groups and led to the dominance of diatoms, including large *Pennales* species^72^. Due to the specificity of available food resources, only consumer species with functionally specialised tasks (*Notholca* spp., *A. ovalis*, *K. testudo*) and species that relied on other food resources such as animal protein (*A. priodonta*) were able to maintain high biomass^70,83^. The network MW was highly fragmented (divided into sub-networks), as indicated by the highest values of the opposing attributes—density and heterogeneity (i.e. the tendency to form concentrated nodes—clusters). A loosening of the network structure, i.e. a decrease in the values of the centrality attributes, increased the importance and the number of taxa communicating with two subnetworks (NBC; Table 3, Fig. 2B). The heterogeneity of the MW network was reflected in strong fragmentation and the formation of large groups with the highest and lowest number of interspecific relationships (Fig. 3B). The largest clusters (CCF ⁓ 1) with many positive and negative relationships contained effective filter feeders, including the cladocerans *Bosmina longiostris* and *Daphnia cucullata*, predatory benthic copepods of the order Harpacticoida, phytophilous rotifers *K. valga* and *Notholca acuminata*, and the protozoan *Arcella discoides*. These observations point to the dynamic character of zooplankton communities in MW due to their taxonomic and functional diversity (feeding strategy, habitat ecology). Taxa with high CCF values (> 0.5) played a crucial role, forming numerous antagonistic relationships (correlation coefficient > − 0.5; Table S2), which were important for maintaining this rich but unstable (due to a very weak second subnetwork) network structure^77^.

Warm winters and small differences in water temperature between seasons significantly affected the rate of physical (decrease in saturation) and biochemical processes (accumulation/immobilisation of organic matter in macrophyte tissues) and reduced phytoplankton production in the warmest reservoir (WW). The above factors weakened interspecific interactions, including negative relationships, and impaired zooplankton network cohesion compared to the colder reservoirs (CW and MW). Network attributes (NCC and NBC) were determined by phytophilous and psammophilous rotifers *Cephalodella* spp., *Monommata maculata* and *Trichocerca intermedia*^70^, littoral cladocerans *Alona* spp., small eurytopic *Chydorus sphaericus* and *B. longirostris*^76^ and protozoa. Thus, populations of zooplankton species characterised by smaller size, lower weight and lower nutrient requirements increased in their biomass. Ejsmont-Karabin et al.^45^ and Kruk et al.^39^ also reported a positive correlation between psammophilous-epiphytic Rotifera and increased and stable water temperature in heated lakes. Other authors observed that the growth of small crustacean species was accelerated by an increase in water temperature^5,26,48,49^. In the present study, the growth of zooplankton was also enhanced by the development of macrophytes, which colonised a large part of the reservoir WW and created a supportive habitat/refuge for the diverse group of Cladocera.

The small littoral cladocerans *Alona* spp. were characterised by the highest values of NCC and NBC (Figs. 2C and 3A) and were responsible for the highest number of individual compounds (NDC), mostly involving negative competitive interactions (Table S2). According to Martín González et al.^84^, species with high levels of NCC and NBC play a special role in zooplankton networks, as the network structure dissolves faster when these species are selectively eliminated. The ratio of positive to antagonistic relationships also plays an important role^77^, and this parameter was not optimal in the WW network. Despite the above, the WW network was characterised by the coexistence of the highest number of rotifer and Cladocera taxa and the highest taxonomic diversity of zooplankton compared to colder reservoirs. Macrophyte habitats probably played an important role in this. Macrophytes promote microbial carbon cycling and increase carbon bioavailability for small zooplankton species when phytoplankton resources are scarce^30^.

In warmer reservoirs, a decline in copepod biomass was followed by the disappearance of most taxa observed in CW. Lower temperature differences and low food availability in warm reservoirs led to long-term interruptions in Copepoda phenology. The lack of distinct seasonal temperature fluctuations in warmer reservoirs may have disrupted the life cycle of copepods (no diapause), resulting in decreased body size and biomass of adult individuals^53,54,85^. According to Santer and Hansen^86^, copepods can skip diapause and develop directly into adults when algal food resources are scarce. This observation was confirmed by the present study, where a decrease in copepod biomass weakened the WW network, as copepods play an important role in predatory interspecific relationships.

### Conclusions

In the natural thermal regime (CW), the network of interspecific interactions was characterised by the highest cohesion and centrality. The CW network had an equal number of positive and negative relationships controlled by eutrophic rotifers (*T. pusilla*, *P. sulcata*, *K. tecta*) and Copepoda. An increase in water temperature in winter and the flattening of the annual temperature gradient reduced centrality and led to the disintegration of the MW and WW networks into clusters (sub-networks). Moderate winters increased the role of ecologically and functionally diverse rotifers (raptorials, phytophiles and predators), which contributed to the heterogeneity of the MW network. In the warmest environment colonised by macrophytes, small littoral cladocerans *Alona* spp. and small psammophilous-epiphytic rotifers (*M. maculata*, *Cephalodella* spp.) formed the most decentralised WW network. Warm winters disrupted the phenology of copepods and reduced their importance in the biocenosis, leading to a decrease in their biomass and the number of antagonistic relationships responsible for network functionality. Network graph modelling adds in an innovative way to the existing knowledge about the functioning of the zooplankton community under the changed thermal conditions. Simultaneously, this method emphasizes the special role of competition and predation in maintaining the durability and resistance of biocenoses, in accordance with the theories of equilibrium in the organization of biocenoses by Armstrong and McGehee^62^ and Levin^63^.

The analysis of network graphs allowed a comprehensive visualisation of changes in plankton communities caused by a temperature increase in surface water reservoirs. The method used clarified the position and role of taxa in the biocenotic network and the ecological mechanisms that are usually difficult to identify and interpret when using conventional structural and multidimensional analyses, especially in in situ studies.

The results on the effects of warm winters and the flattening of the annual water temperature amplitude on the zooplankton network could be a projection of expected global changes. These effects are particularly important in water reservoirs that are subject to anthropogenic pressures and where changes in the thermal regime may influence future ecosystem services.

## Methods

### Study area

The study was conducted in three artificial reservoirs (CH1, PN, WI) near the Bełchatów open-cast lignite mine in central Poland (51°24′43.6″ N; 19°26′32.9″ E). The reservoirs serve as sediment ponds of drainage dewatering system for the opencasts Bełchatów and Szczerców (Fig. S2). Their main function is to reduce suspended matter through sedimentation^72,74,87^. These flow-through basins (with an estimated residence time of 16 h) have a similar structure, shape, area (7.1–8.2 ha) and depth (1.7–2.7 m)^72,88^. The feedwater comes from different depths and differs in temperature.

Zooplankton were taken from three artificial reservoirs in the Bełchatów-Szczerców open-cast coal mine (central Poland), which are fed with water from different depths, including geothermal springs. The temperature of the feed water is therefore different. In the studied geological region, the availability of geothermal water is determined by deposits from the Early Jurassic, where the water table has a stable temperature of 40–50 °C ^89^. As a result, the studied reservoirs differ significantly in mean annual temperature and annual temperature gradient, especially in winter. The structures of the plankton communities could be compared in situ under different thermal conditions, as the studied ecosystems have similar limnological and hydrological parameters and are used in a controlled manner.

Reservoir CH1 is filled with atmospheric water, meltwater and capillary water with a temperature similar to the air temperature^87,90,91^. Therefore, reservoir CH1 represents the natural seasonal variations of temperature of shallow waters in temperate climate (Fig. 4). The reservoir WI is fed by water from a deep drainage well (up to 350 m) with a stable temperature of > 30 °C, which is characteristic of geothermal wells^89,90^. The PN reservoir is mainly fed by deep drainage wells as well as by surface runoff from a coal deposit. Therefore, the temperature of the feedwater in reservoir PN corresponds to the middle range of values describing the feedwater in reservoirs CH1 and WI. The water is transported to the reservoirs via open concrete channels with a length of 1–1.5 km, which reduces the differences in water and air temperature. The water transported to the warmest reservoir (WI) has a temperature of about 16–18 °C in winter (when the air temperature is 0–4 °C), which means that the difference between the compared reservoirs is greatest in the coldest season (Fig. 4). Furthermore, in mild winters the growth period of aquatic macrophytes in the reservoir is prolonged WI, and macrophytic vegetation colonises both the littoral zone and large parts of the water surface (mainly *Nuphar lutea* L.) throughout the year.

The influence of water temperature on interactions between zooplankton species was analysed in three winter temperature scenarios: cold winters (CW < 6 °C)—reservoir CH1, moderate winters (MW = 6–10 °C)—reservoir PN, and warm winters (WW > 10 °C)—reservoir WI.

### Sampling and analytical procedure

Zooplankton were sampled monthly, between January and December in 2014 and 2015, and between June and September in 2016. In each reservoir, samples were collected at three locations in the middle, in the littoral zone and near the filter zone (see in Goździejewska et al.^67^). Samples were collected with a 5-L sampler at an estimated depth of 1 m below the water surface. During the field study, a total of 252 zooplankton samples (84 samples from each of the three reservoirs) were collected. The sampled material (20 l) was filtered through a 30 μm mesh plankton net and preserved with a 4% formalin solution. Zooplankton were identified to the lowest possible taxonomic level (with the exception of juvenile Copepoda stages) under a Zeiss AXIO Imager microscope using the methods see^70,76,85,92,93^. The abundance of zooplankton (ind. l^−1^) was determined in quantitative analyses using a Sedgewick-Rafter counting chamber. Zooplankton biomass (mg l^−1^) was determined using the methods see^94,95^. Diversity (Shannon index, *H*′), species evenness (Pielou index, *J*′) and similarity of zooplankton communities (Jaccard coefficient, *P*′) were analysed using MVSP 3.22 software^96^.

The physical and chemical parameters of the water were analysed at a single point in the middle of each reservoir during each sampling. Water temperature (°C), pH and dissolved oxygen (DO, mg l^−1^) were measured using the YSI 6600 V2 multi-parameter water quality probe. Water transparency (SD, m) was measured with a Secchi disc. In the laboratory, water samples were analysed for colour (Hazen scale), turbidity (NTU), nitrate (NO_3_-N, mg l^−1^), ammonium (NH_4_-N, mg l^−1^), total nitrogen (TN, mg l^−1^), orthophosphate (PO_4_-P, mg l^−1^), total phosphorus (TP, mg l^−1^), total organic carbon (TOC, mg l^−1^), chlorophyll *a* (Chl *a*, µg l^−1^), inorganic suspended solids (SSmin, mg l^−1^), organic suspended solids (SSorg, mg l^−1^), total suspended solids (SStot, mg l^−1^), and iron (Fe, mg l^−1^). The hydrochemical analyses were carried out in accordance with APHA guidelines^97^.

### Statistical and network analyses

Overall differences in physical and chemical parameters of water and zooplankton between the analysed thermal classes were determined by one-way ANOVA (f, *P* ≤ 0.05) and Tukey's HSD test. The non-parametric Kruskal–Wallis test (H, *P* ≤ 0.05) was used to determine differences in zooplankton biomass between thermal classes (Statistica 13.0 for Windows, Statsoft, Tulsa). Spearman's rank correlation analysis (*P* < 0.05) was used to test for correlations between temperature and zooplankton species richness, and between temperature and the other physical and chemical variables of water.

To test the assumption that the studied reservoirs can serve as a model for studying the influence of thermals on zooplankton assemblage, we performed a feature importance analysis. To show that water temperature variability is the dominant differentiating factor in the three reservoirs studied, we carried out a procedure to evaluate the importance (F score) of variables for these waters, which were treated as three thermal classes. We used a predictive model, eXtremeGradientBoosting (XGBoost), based on the boosting technique. It assumes random selection of interactions between factors and uses boosting of individual variables to obtain a model with the highest accuracy. In this way, we obtain a ranking of variables based on their involvement in the construction of the most accurate model^98^. We adopted a code from the Kaggle notebook ’Ensembles and Model Stacking’^99^.

Graph theory was applied to compare the zooplankton network parameters in three thermal classes and to determine the importance of individual species and interspecific interactions in these networks. Interactions between zooplankton species in three thermal classes were analysed in the Cytoscape platform (http://www.cytoscape.org/) using the MetScape and NetworkAnalyzer applications to determine correlations between data points. The data were normalised by autoscaling. The correlation matrix was calculated using the Correlation Calculator 1.01 programme (University of Michigan).

In graph theory, the connections (edges) between species (nodes) are studied by analysing the parameters of the entire network and determining the extent to which the attributes of each node and edge affect the network and centrality measures^100^. An undirected graph was constructed to identify all positive and negative interactions between zooplankton species in three thermal classes. Positive interactions denoted co-occurrence patterns or mutualistic relationships between the biomass of zooplankton taxa, while negative interactions denoted predatory or competitive relationships^37,40^. The ranges of values of the correlation coefficients for the edges were set to be significant at* P* ≤ 0.05 for the sample size in each thermal class. The edge-weighted layout of the embedded springs was used with correlation coefficients as weights and weight-based heuristics. The absolute values of correlation coefficients between nodes were used as weights. In weighted graphs, the distance between nodes is defined as the sum of the weights^101^. The zooplankton network in the three thermal classes was compared using the main network attributes used in ecological studies, including number of neighbours, nearest path, clustering coefficient, network centralisation, network density and network heterogeneity^37,40,102^. Four common attributes of nodal centrality were used to determine the importance of zooplankton taxa in three thermal classes: node degree centrality (NDC)^102^, node closeness centrality (NCC)^103^, node betweenness centrality (NBC)^104^ and the clustering coefficient (CCF).

NCC is a measure of how fast information, defined here by significant correlations between taxa, spreads from one particular species to others in the network^105^. The higher the closeness centrality, the more important the biomass of the zooplankton species is for organising other interactions in the biocoenosis network^106^. NBC refers to the extent to which a particular taxon contributes to network cohesion by communicating with other clusters (subnets). The global network clustering coefficient measures the degree to which the nodes (species) in the graph tend to cluster together. Each taxon has an individual (local) clustering coefficient (CCF), which is the ratio between the actual number of connections between a given taxon and its nearest neighbours and the possible number of connections in a complete graph if all possible connections (100%) are present in a given cluster^107^.

### Supplementary Information

Supplementary Information.

## Data Availability

The datasets generated during and analyzed during the current study are not publicly available due to rules established by the Project Funder but are available from the corresponding author on reasonable request.
