# Supplementary material for: The response of zooplankton network indicators to winter water warming using shallow artificial reservoirs as model case study
Source: Sci Rep. 2023 Oct 21;13:18002. doi: 10.1038/s41598-023-45430-7 (PMC10590368; doi:10.1038/s41598-023-45430-7)
Supplement: Supplementary file 1 — Supplementary Information. [file 41598_2023_45430_MOESM1_ESM.pdf]

Supplement of:

The response of zooplankton network indicators to winter water warming using shallow artificial reservoirs as model case study

Anna Maria Goździejewska and Marek Kruk

Correspondence to: Anna Maria Goździejewska (gozdzik@uwm.edu.pl)

Table S1. Species composition, biomass (μg l^{-1}; mean ± SD), and zooplankton frequency (%) in individual thermal classes. Values with different superscripts differ significantly across reservoirs in the Kruskal–Wallis non-parametric test ($P \leq 0.05$).

**Taxa**	**COLD**			**MIDDLE**			**WARM**			**Kruskal-Wallis**
	Biomass (μg l^{-1})		Freq	Biomass (μg l^{-1})		Freq	Biomass (μg l^{-1})		Freq	
	$\bar{x}$	±SD	(%)	$\bar{x}$	±SD	(%)	$\bar{x}$	±SD	(%)	*P*
**Rotifera**										
Anuraeopsis fissa	0.007	0.019	13	-	-	-	-	-	-	>0.05
Ascomorpha ovalis	0.266^{a}	0.295	54	0.580^{b}	0.392	75	0.121^{a}	0.225	25	0.0001
Ascomorpha saltans	-	-	-	0.008	0.041	4	-	-	-	>0.05
Asplanchna priodonta	28.71^{a}	27.83	58	13.12^{b}	22.23	29	-	-	-	0.037
Brachionus angularis	0.414^{a}	0.577	42	0.065^{b}	0.235	8	0.065^{b}	0.177	13	0.005
Brachionus calyciflorus	0.826	2.035	17	0.092	0.450	4	-	-	-	>0.05
Brachionus diversicornis	0.057	0.277	4	-	-	-	0.057	0.277	4	>0.05
Brachionus falcatus	-	-	-	-	-	-	0.057	0.277	4	>0.05
Brachionus leydigii	0.497	2.434	4	1.49	3.06	25	-	-	-	>0.05
Brachionus quadridentatus	-	-	-	0.138	0.678	4	0.277	0.937	8	>0.05
Cephalodella auriculata	-	-	-	0.013	0.042	8	0.013	0.061	4	>0.05
Cephalodella forficula	-	-	-	-	-	-	0.015	0.072	4	>0.05
Cephalodella gibboides	0.100	0.358	8	0.111	0.264	17	0.262	0.683	21	>0.05
Cephalodella psammophila	-	-	-	0.038	0.106	13	0.045	0.096	21	>0.05
Cephalodella sp.	0.013^{a}	0.042	8	0.088^{ab}	0.124	38	0.169^{b}	0.199	58	0.001
Colurella colurus	0.023^{a}	0.046	21	0.103^{b}	0.114	54	0.107^{b}	0.134	46	0.006
Colurella uncinata	0.018	0.064	8	0.012	0.040	8	0.047	0.090	25	>0.05
Conochilus unicornis	0.065	0.319	4	0.022	0.106	4	-	-	-	>0.05
Dicranophorous sp.	0.138	0.493	8	0.092	0.449	4	-	-	-	>0.05
Dipeuchlanis propatula	-	-	-	-	-	-	0.033	0.163	4	>0.05
Encentrum sp.	-	-	-	-	-	-	0.016	0.080	4	>0.05
Euchlanis contorta	-	-	-	0.133	0.652	4	0.665	1.879	13	>0.05
Euchlanis dapidula	-	-	-	0.266	0.902	8	0.399	1.079	13	>0.05
Euchlanis dilatata	0.146^{a}	0.714	4	0.073^{a}	0.357	4	1.457^{b}	1.835	46	0.0001
Euchlanis lyra	-	-	-	-	-	-	0.292	0.843	13	>0.05
Filinia longiseta	0.437	0.612	42	0.125	0.337	17	0.166	0.240	33	>0.05
Hexarthra mira	0.561	1.250	21	-	-	-	0.280	0.685	17	>0.05
Kellicottia longispina	0.016	0.054	8	-	-	-	-	-	-	>0.05
Keratella cochlearis	0.407^{a}	0.089	100	0.110^{b}	0.125	50	0.252^{b}	0.223	67	0.0000
Keratella paludosa	-	-	-	0.008	0.041	4				>0.05
Keratella quadrata	1.55^{a}	1.05	79	0.529^{b}	0.781	38	0.564^{b}	0.734	46	0.0001
Keratella tecta	0.258	0.218	63	0.161	0.202	46	0.129	0.175	46	>0.05
Keratella testudo	0.012	0.060	4	0.098	0.223	21	-	-	-	>0.05
Keratella ticinensis	0.005	0.024	4	-	-	-	-	-	-	>0.05
Keratella valga	0.245	0.363	38	0.140	0.234	33	0.140	0.286	25	>0.05
Lecane arcula	-	-	-	-	-	-	0.002	0.011	4	>0.05
Lecane bulla	0.033^{ab}	0.110	8	0.016^{a}	0.080	4	0.228^{b}	0.344	38	0.004
Lecane closterocerca	-	-	-	0.004	0.021	4	0.030	0.057	25	>0.05
Lecane cornuta	-	-	-	-	-	-	0.029	0.142	4	>0.05
Lecane flexilis	0.004	0.021	4	0.013	0.047	8	-	-	-	>0.05
Lecane hamata	0.005	0.024	4	-	-	-	-	-	-	>0.05
Lecane imbricata	-	-	-	0.002	0.011	4	-	-	-	>0.05
Lecane inermis	-	-	-	0.005	0.018	8	-	-	-	>0.05
Lecane levistyla	-	-	-	-	-	-	0.011	0.056	4	>0.05
Lecane ludwigii	-	-	-	-	-	-	0.022	0.073	8	>0.05
Lecane luna	0.021	0.105	4	-	-	-	0.236	0.371	33	>0.05
Lecane lunaris	0.032	0.086	13	0.021	0.104	4	0.106	0.182	29	>0.05
Lecane quadridentata	-	-	-	-	-	-	0.160	0.451	13	>0.05
Lecane tryphema	-	-	-	0.006	0.032	4	0.006	0.032	4	>0.05
Lepadella ovalis	0.026	0.060	17	0.066	0.123	29	0.086	0.132	38	>0.05
Lepadella patella	0.007	0.036	4	0.007	0.036	4	-	-	-	>0.05

Lepadella rhomboides	-	-	-	-	-	-	0.051	0.146	13	>0.05
Monommata maculata	0.031^{a}	0.153	4	0.281^{b}	0.577	25	0.281^{b}	0.693	21	0.041
Mytilina mucronata				0.044	0.217	4	0.133	0.359	13	>0.05
Notholca acuminata	0.479	1.024	21	0.752	1.278	33	0.205	0.554	13	>0.05
Notholca labis				0.028	0.096	8	-	-	-	>0.05
Notholca squamula	0.088^{a}	0.260	13	0.480^{b}	0.367	71	0.013^{a}	0.062	4	0.0000
Notomata sp.				0.013	0.061	4	-	-	-	>0.05
Polyarthra longiremis	1.64^{a}	0.490	96	0.835^{b}	0.755	67	0.683^{b}	0.787	54	0.0000
Polyarthra major	0.042	0.204	4	-	-	-	0.042	0.204	4	>0.05
Polyarthra vulgaris	0.443^{a}	0.496	46	-	-	-	0.014^{b}	0.068	4	0.0006
Pompholyx complanata	0.013	0.050	8	0.010	0.035	8	0.010	0.048	4	>0.05
Pompholyx sulcata	0.157	0.257	33	-	-	-	-	-	-	>0.05
Proales sp.	0.292	0.550	25	0.417	0.929	21	0.458	0.658	38	>0.05
Scaridium longicaudum	-	-	-	-	-	-	0.069	0.177	17	>0.05
Squatinella rostrum	-	-	-	-	-	-	0.020	0.067	8	>0.05
Synchaeta sp.	6.36^{a}	4.56	75	4.73^{a}	4.98	54	0.740^{b}	1.473	21	0.0001
Testudinella patina				0.415^{a}	0.774	25	0.035^{b}	0.169	4	0.037
Trichocerca bidens	-	-	-	-	-	-	0.023	0.112	4	>0.05
Trichocerca elongata	-	-	-	-	-	-	0.017	0.084	4	>0.05
Trichocerca insignis	-	-	-	0.100	0.188	25	-	-	-	>0.05
Trichocerca intermedia	-	-	-	0.003	0.012	8	0.007	0.020	13	>0.05
Trichocerca musculus	-	-	-	0.021	0.057	13	0.021	0.076	8	>0.05
Trichocerca myersi	-	-	-	-	-	-	0.057	0.130	17	>0.05
Trichocerca porcellus	-	-	-	0.042	0.151	8	0.028	0.095	8	>0.05
Trichocerca pusilla	0.057	0.115	21	0.005	0.023	4	0.014	0.051	8	>0.05
Trichocerca rattus	-	-	-	-	-	-	0.014	0.069	4	>0.05
Trichocerca scipio	-	-	-	0.005	0.023	4	-	-	-	>0.05
Trichocerca similis	-	-	-	0.011	0.052	4	0.011	0.052	4	>0.05
Trichocerca stylata	0.038	0.128	8	-	-	-	-	-	-	>0.05
Trichocerca tenuior	-	-	-	0.058	0.132	21	0.029	0.066	17	>0.05
Trichocerca tigris	-	-	-	0.061	0.173	13	0.061	0.150	17	>0.05
Trichocerca vernalis	-	-	-	-	-	-	0.009	0.044	4	>0.05
Trichocerca weberi	-	-	-	-	-	-	0.005	0.023	4	>0.05
Trichotria pocillum	0.049	0.164	8	0.073	0.197	13	0.121	0.241	21	>0.05
Trichotria tetractis	-	-	-	0.030	0.148	4	0.182	0.386	21	>0.05
**Cladocera**										
Acroperus harpae	-	-	-	0.417	2.041	4	0.833	2.823	8	>0.05
Alona affinis	-	-	-	1.25	4.48	8	0.833	2.823	8	>0.05
Alona costata	-	-	-	0.417	2.041	4	-	-	-	>0.05
Alona guttata	-	-	-	-	-	-	1.25	4.48	8	>0.05
Alona protzi	-	-	-	-	-	-	0.417	2.041	4	>0.05
Alona quadrangularis	-	-	-	2.92	8.90	17	0.792	2.686	8	>0.05
Alona rectangula	-	-	-	-	-	-	0.417	2.041	4	>0.05
Alonella nana	-	-	-	-	-	-	0.417	2.041	4	>0.05
Bosmina longirostris	14.13^{ab}	20.77	46	13.96^{a}	14.18	58	3.38^{b}	7.26	21	0.0185
Ceriodaphnia quadrangula	-	-	-	2.08	5.88	13	-	-	-	>0.05
Chydorus sphaericus	-	-	-	0.208	1.021	4	1.54	2.48	29	>0.05
Daphnia cucullata	213.1^{a}	115.6	88	69.58^{b}	108.25	46	50.83^{b}	63.86	46	0.0000
Eurycercus lamellatus	-	-	-	-	-	-	4.17	20.41	4	>0.05
Graptoleberis testudinaria	-	-	-	-	-	-	3.75	10.13	13	>0.05
Ilyocryptus agilis	1.67	8.16	4	-	-	-	-	-	-	>0.05
Leptodora kindtii	20.83	50.90	17	-	-	-	-	-	-	>0.05
Pleuroxus truncatus	-	-	-	-	-	-	2.08	7.21	8	>0.05
Scapholeberis mucronata	-	-	-	-	-	-	1.67	8.16	4	>0.05
**Copepoda**										
Acanthocyclops robustus	1.04	5.10	4	-	-	-	-	-	-	>0.05
Cryptocyclops bicolor	1.25	6.12	4	-	-	-	-	-	-	>0.05
Cyclops scutifer	1.67	8.16	4	-	-	-	-	-	-	>0.05
Cyclops strenuus	7.50	21.72	13	-	-	-	-	-	-	>0.05
Cyclops vicinus	9.17	22.63	17	-	-	-	-	-	-	>0.05
Diacyclops crassicaudis	-	-	-	5.00	19.11	8	1.25	6.12	4	>0.05

Eucyclops macruroides	1.25	6.12	4	-	-	-	-	-	-	>0.05
Eucyclops serrulatus	-	-	-	1.25	6.12	4	-	-	-	>0.05
Eucyclops speratus	-	-	-	5.00	11.42	17	-	-	-	>0.05
Eudiaptomus graciloides	11.67	34.35	13	-	-	-	-	-	-	>0.05
Harpacticoida	1.88	5.07	13	0.625	3.062	4	3.75	7.97	21	>0.05
copepodites	27.25[a]	11.38	100	8.33[b]	9.63	50	7.08[b]	8.59	54	0.0000
Microcyclops varicans	2.50	8.97	8	0.833	4.082	4	-	-	-	>0.05
nauplii	2.13	0.689	100	1.604	0.895	96	1.56	1.01	92	>0.05
Thermocyclops crassus	20.00[a]	30.22	38	-	-	-	0.104[b]	0.510		0.0036
Paracyclops fimbriatus	-	-	-	-	-	-	0.833	4.082	4	>0.05
**Protozoa**										
Arcella discoides	0.010[a]	0.025	17	0.010[a]	0.021	21	0.106[b]	0.065	83	0.0000
Centropyxis aculeata	0.008[a]	0.019	17	0.006[a]	0.017	13	0.042[b]	0.041	63	0.0000
Codonella cratera	0.098[a]	0.062	75	0.015[b]	0.028	25	0.021[b]	0.039	29	0.0000
Difflugia acuminata	-	-	-	-	-	-	0.013	0.045	8	>0.05
Difflugia limnetica	-	-	-	-	-	-	0.004	0.020	4	>0.05
Difflugia lobostoma	0.088[ab]	0.133	33	0.021[a]	0.051	17	0.100[b]	0.114	54	0.004
Difflugia pyriformis	-	-	-	-	-	-	0.017	0.048	13	>0.05

Table S2. The most important relationships between zooplankton species in the compared thermal classes.

Thermal class	Ralationships between taxa		
COLD	Negative	*Brachionus calyciflorus* - *Keratella tecta*	-0.671
		Keratella valga - *Pompholyx sulcata*	-0.638
		Harpacticoida - nauplii	-0.591
		Bosmina longirostris - *Keratella quadrata*	-0.584
		nauplii - *Keratella tecta*	-0.556
		Difflugia lobostoma - *Filinia longiseta*	-0.529
		Keratella valga - *Trichocerca pusilla*	-0.517
		Pompholyx sulcata - *Trichocerca pusilla*	-0.484
		Cyclops vicinus - *Trichocerca pusilla*	-0.473
		Keratella cochlearis - *Synchaeta* spp.	-0.471
		Brachionus calyciflorus - *Filinia longiseta*	-0.461
		Keratella tecta - *Lepadella* spp.	-0.457
		Difflugia lobostoma - *Brachionus calyciflorus*	-0.457
		Thermocyclops crassus - *Proales* sp.	-0.445
		Cryptocyclops bicolor - *Codonella cratera*	-0.435
		Cryptocyclops bicolor - *Keratella cochlearis*	-0.431
		Codonella cratera - *Brachionus calyciflorus*	-0.424
		Cryptocyclops bicolor - *Synchaeta* spp.	-0.419
		Keratella cochlearis - *Trichocerca pusilla*	-0.413
		Keratella quadrata - *Keratella valga*	-0.401
	Positive	*Ascomorpha ovalis* - *Notholca squamula*	0.837
		Keratella tecta - *Pompholyx sulcata*	0.773
		Polyarthra longiremis - *Synchaeta* spp.	0.759
		Microcyclops varicans - *Centropyxis aculeata*	0.747
		Keratella tecta - *Keratella valga*	0.713
		Filinia longiseta - *Hexarthra mira*	0.657
		Harpacticoida - *Colurella colurus*	0.654
		Brachionus angularis - *Keratella cochlearis*	0.616
		copepodites - *Thermocyclops crassus*	0.610
		Lepadella spp. - *Notholca squamula*	0.607
		Brachionus calyciflorus - *Polyarthra longiremis*	0.603
		Brachionus calyciflorus - *Keratella valga*	0.588
		nauplii- *Keratella valga*	0.584
		Cryptocyclops bicolor - *Polyarthra longiremis*	0.567
		Lepadella spp. - *Pompholyx sulcata*	0.564
		nauplii - *Pompholyx sulcata*	0.554
		Brachionus calyciflorus - *Polyarthra vulgaris*	0.552
		Codonella cratera - *Polyarthra longiremis*	0.533
		Daphnia cucullata - *Keratella cochlearis*	0.532
		Bosmina longirostris - nauplii	0.524
MIDDLE	Negative	*Brachionus calyciflorus* - *Synchaeta* spp.	-0.821
		Brachionus calyciflorus - *Keratella valga*	-0.754
		Bosmina longirostris - *Keratella testudo*	-0.701
		Keratella valga - *Synchaeta* spp.	-0.689
		Keratella tecta - *Trichocerca tigris*	-0.677
		Bosmina longirostris - *Keratella tecta*	-0.636
		Chydorus sphaericus - *Notholca acuminata*	-0.632
		Chydorus sphaericus - *Euchlanis* spp.	-0.592
		Euchlanis spp. - *Notholca acuminata*	-0.591
		Ascomorpha ovalis - *Synchaeta* spp.	-0.586

		Ascomorpha ovalis - *Brachionus calyciflorus*	-0.571
		Codonella cratera - *Keratella valga*	-0.57
		Keratella quadrata - *Keratella testudo*	-0.562
		Chydorus sphaericus - *Microcyclops varicans*	-0.553
		Microcyclops varicans - *Notholca acuminata*	-0.551
		Polyarthra longiremis - *Trichocerca pusilla*	-0.54
		Proales sp. - *Trichocerca tigris*	-0.523
		Asplanchna priodonta - *Keratella testudo*	-0.519
		Bosmina longirostris - *Keratella quadrata*	-0.518
		Ascomorpha ovalis - *Keratella valga*	-0.507
	**Positive**	*Polyarthra longiremis* - *Trichocerca tigris*	0.961
		Bosmina longirostris - *Ascomorpha ovalis*	0.932
		Asplanchna priodonta - *Keratella valga*	0.907
		Brachionus calyciflorus - *Keratella tecta*	0.844
		Brachionus calyciflorus - *Filinia longiseta*	0.828
		Arcella discoides - *Notholca acuminata*	0.821
		Filinia longiseta - *Synchaeta* spp.	0.782
		Chydorus sphaericus - *Arcella discoides*	0.77
		kopepodit - *Microcyclops varicans*	0.721
		Daphnia cucullata - Harpacticoida	0.697
		Ascomorpha ovalis - *Keratella testudo*	0.697
		Microcyclops varicans - *Arcella discoides*	0.677
		Bosmina longirostris - *Synchaeta* spp.	1
		Keratella tecta - *Synchaeta* spp.	0.659
		Arcella discoides - *Euchlanis* spp.	0.647
		Bosmina longirostris - *Brachionus calyciflorus*	0.645
		Bosmina longirostris - *Keratella valga*	0.616
		Daphnia cucullata - *Synchaeta* spp.	0.609
		Codonella cratera - *Asplanchna priodonta*	0.605
		Ascomorpha ovalis - *Keratella quadrata*	0.599
**WARM**	**Negative**	*Cephalodella* spp. - *Polyarthra vulgaris*	-0.582
		Alona quadrangularis - *Ascomorpha ovalis*	-0.397
		Centropyxis aculeata - *Trichocerca intermedia*	-0.337
		Alona quadrangularis - *Hexarthra mira*	-0.32
		Monommata maculata - *Trichocerca intermedia*	-0.312
		nauplii - *Centropyxis aculeata*	-0.305
		Alona quadrangularis - *Chydorus sphaericus*	-0.29
		Difflugia lobostoma - *Euchlanis* spp.	-0.289
		Alona quadrangularis - *Notholca squamula*	-0.282
	**Positive**	*Brachionus angularis* - *Polyarthra vulgaris*	0.779
		Alona quadrangularis - *Centropyxis aculeata*	0.67
		Chydorus sphaericus - *Trichocerca intermedia*	0.67
		Bosmina longirostris - *Notholca acuminata*	0.658
		Brachionus angularis - *Cephalodella* spp.	0.651
		Testudinella patina - *Trichocerca tenuior*	0.646
		Notholca squamula - *Synchaeta* spp.	0.635
		Lepadella spp. - *Notholca acuminata*	0.591
		Euchlanis spp. - *Hexarthra mira*	0.57
		Chydorus sphaericus - *Monommata maculata*	0.552
		Centropyxis aculeata - *Ascomorpha ovalis*	0.521
		Ascomorpha ovalis - *Trichocerca tenuior*	0.441
		Synchaeta spp. - *Trichocerca tenuior*	0.405
		Difflugia lobostoma - *Hexarthra mira*	0.394
		Cephalodella spp. - *Polyarthra longiremis*	0.384
		Bosmina longirostris - *Monommata maculata*	0.381
		Codonella cratera - *Trichocerca intermedia*	0.375

		Colurella colurus - *Lepadella* spp.	0.372
		Difflugia lobostoma - *Lecane* spp.	0.363
		Keratella quadrata - *Polyarthra longiremis*	0.353

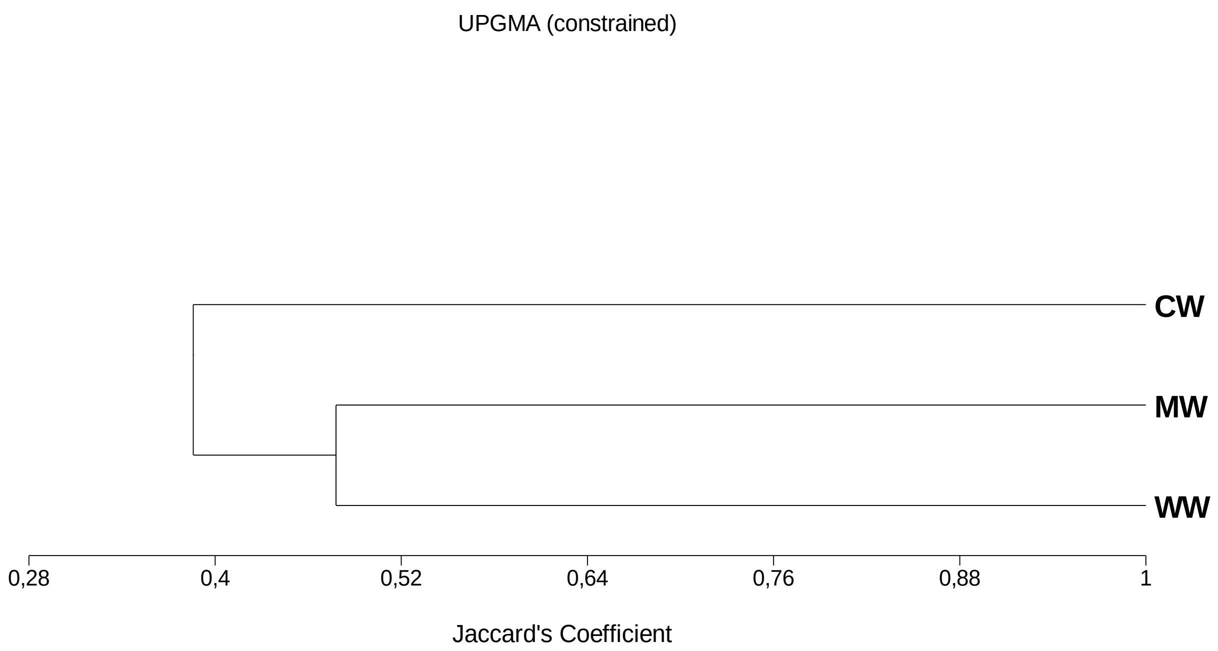

Figure S1. Faunistic similarity of zooplankton communities in the compared thermal classes.

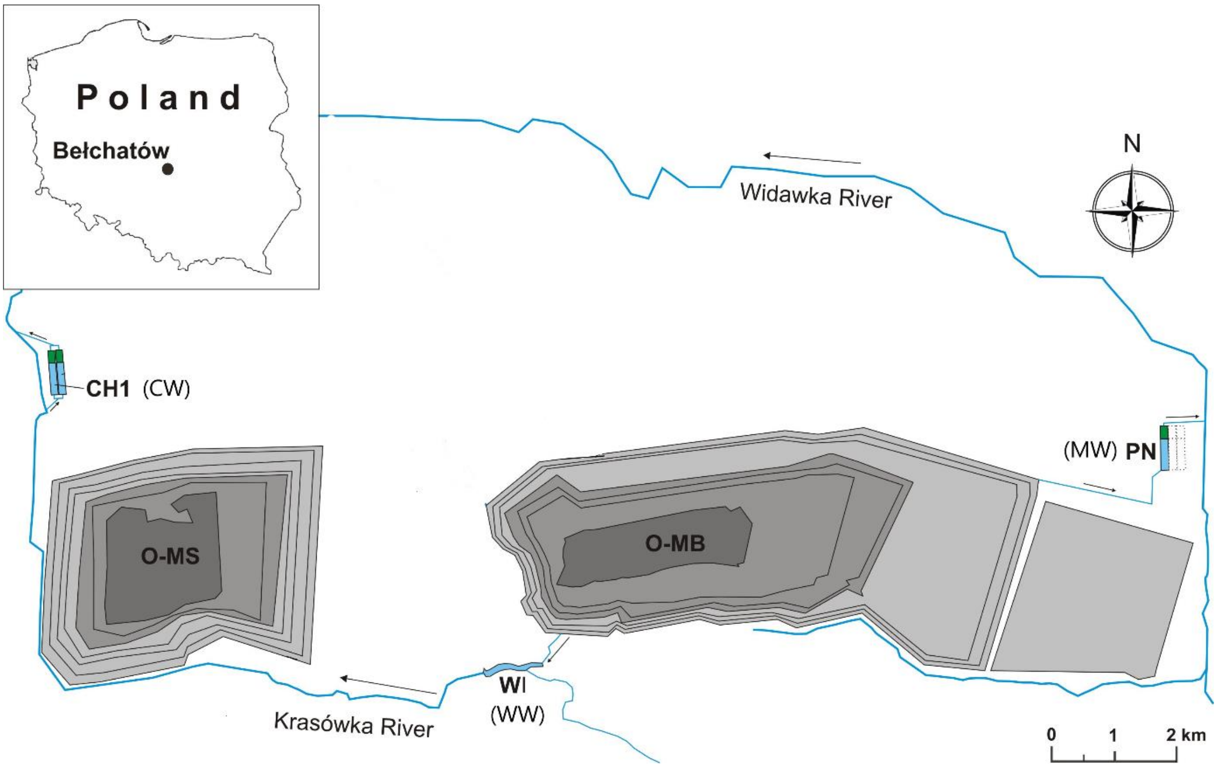

Figure S2. Location of the study area. Abbreviations: O-MB – opencast mine in Bełchatów, O-MS – opencast mine in Szczerców; reservoir CH1 represents CW class, reservoir PN represents MW class; reservoir WI represents the WW class. Modified, see Goździejewska and Kruk (2022).
